# Efficacy of Intravitreal Injection of Filtered Modified Low-Dose Triamcinolone Acetonide and Ranibizumab on Pseudophakic Cystoid Macular Edema

**DOI:** 10.3389/fmed.2022.777549

**Published:** 2022-02-16

**Authors:** Farheen Tariq, Yanfen Wang, Bo Ma, Yidan He, Shu Zhang, Ling Bai

**Affiliations:** ^1^Department of Ophthalmology, The Second Affiliated Hospital of Xi'an Jiaotong University, Xi'an, China; ^2^Department of Ophthalmology, The No.4 Hospital of Xi'an, Xi'an, China; ^3^Experimental Teaching Center for Clinical Skills and Department of Geriatric Medicine, The Second Affiliated Hospital of Xi'an Jiaotong University, Xi'an, China

**Keywords:** triamcinolone acetonide, ranibizumab, pseudophakic eye, cystoid macular edema, intravitreal injection

## Abstract

**Purpose:**

To compare the visual and morphological effects between intravitreal injection of filtered modified 2 mg triamcinolone acetonide (TA) and 0. 5 mg ranibizumab in patients with pseudophakic cystoid macular edema (PCME).

**Methods:**

A retrospective, interventional study was conducted from January 2015 to February 2020 involving patients with PCME after uneventful cataract surgery. A total of 25 patients (25 eyes) with PCME received an intravitreal injection of 0.22 μm filtered modified 2 mg TA, while 15 patients (15 eyes) received 0.5 mg ranibizumab injection. Central macular thickness (CMT), best-corrected visual acuity (BCVA), intraocular pressure (IOP), times of repeated injections, and other side effects were observed at 2 weeks, 1 month, 3 months, and 6 months after injection; then, the data were compared with preinjection information in each group and between the two groups.

**Results:**

Both the TA and ranibizumab intravitreal injection can achieve improved BCVA and reduced CMT in patients with PCME (*P* < 0.05), with a trend toward greater improvement in the TA group, but the difference was only significant at 3 months (*P* < 0.05). IOP was in the normal range without any significant difference (*P* > 0.05). Thirty-three percent of patients in the ranibizumab group required repeated intravitreal injection compared to 4% in the TA group. Further stratified analysis showed that the better therapeutic effect of the TA group at 3 months after injection only existed in patients with diabetes mellitus (DM), while not in patients without DM. There was no repeat injection in the TA group and 12.5% in the ranibizumab group for patients without DM, while 16.7% in the TA group and 57.1% in the ranibizumab group required repeated injection for patients with DM, which had a significant difference (*P* < 0.05).

**Conclusion:**

Intravitreal injection of filtered modified 2 mg TA is safe, effective, and an inexpensive alternative to antivascular endothelial growth factor (anti-VEGF) agents for patients with PCME, especially for patients concurrently with DM. A large number of clinical randomized controlled studies along with long-term follow-up observations are needed.

## Introduction

Pseudophakic cystoid macular edema (PCME), also known as Irvine–Gass syndrome (IGS), remains to be a remarkable cause of compromised vision recovery after cataract surgery. Despite the success of contemporary phacoemulsification, non-steroidal anti-inflammatory drugs and glucocorticoid eye drops significantly reduce the incidence; 0.1–8% of patients still have significant clinical manifestations of PCME (1). Due to the lack of large-scale randomized controlled trials for its treatment, there is still no guideline or generally accepted expert opinion for PCME treatment (2).

Recently, large randomized controlled clinical trials have shown that intravitreous injections of anti-vascular endothelial growth factor (anti-VEGF) agents such as bevacizumab (BVB) (3), ranibizumab (4, 5), and aflibercept (6, 7) were efficient for diabetic macular edema (DME) with improved visual results than laser photocoagulation, which was the previous standard treatment for DME. However, current findings have recommended that these agents may not be as useful for PCME (2).

Triamcinolone acetonide (TA) is a synthetic long-acting glucocorticoid with strong and lasting anti-inflammatory and anti-VEGF effect. Many studies have confirmed that intravitreal injection of TA (IVTA) for PCME is economical and effective, but its side effects of intraocular pressure (IOP) rise cannot be avoided (8, 9). Most studies have used 4 mg/1 ml of IVTA for the treatment of PCME (10–12). Forty to fifty percent of rates rise in IOP have been reported after 4 mg of IVTA (13).

Several researchers (14, 15) have compared the therapeutic effects of anti-VEGF agents and IVTA in DME. But, there was no comparison between their usage in PCME, especially the filtered modified low-dose TA. This study aims to compare the effectiveness and safety of intravitreal injection of filtered modified 2 mg TA and 0.5 mg ranibizumab in PCME, by the index of best-corrected visual acuity (BCVA), central macular thickness (CMT), IOP, times of repeated injections, and complications.

## Methods and Objectives

### Objectives

This was an interventional, retrospective study of eyes with PCME after uneventful cataract surgery from January 2015 to February 2020. The clinical records of 23 males and 17 females were reviewed, including 13 cases of diabetes mellitus (DM) and the average age was 69.20 ± 10.20 years. Fifteen males (15 eyes) and 10 females (10 eyes) were included in the TA injection group, while eight males (eight eyes) and seven females (seven eyes) were included in the ranibizumab group. PCME occurred at 3 weeks to 5 months after cataract surgery. All the patients have received phacoemulsification combined with posterior chamber intraocular lens (IOL) implantation. The surgery went smoothly without any intraoperative complications. After the operation, tobramycin-dexamethasone and pranoprofen eye drops were used as routine. All the patients were suffering from symptomatic macular edema and local treatment with non-steroidal anti-inflammatory drugs or glucocorticoids had no obvious effect for at least 1 month after treatment. The inclusion criteria of macular edema were: CMT increased by more than 40% after cataract surgery confirmed by optical coherence tomography (OCT) (16). Exclusion criteria were as follows:

- Intraocular pressure >21 mm Hg or history of glaucoma before the operation.
- Patients having previous intraocular surgery.
- Systematic use of anti-VEGF agents previously.
- Patients having a history of laser photocoagulation of retina.
- Intraoperative and postoperative complications of cataract surgery other than PCME such as posterior capsule rupture, vitreous hemorrhage, and retinal detachment.
- History of eye or general injury.
- History of eye disease such as uveitis, retinal vein occlusion, age-related macular degeneration, and epiretinal membrane.
- Diabetic patients, who preoperatively had macular edema and the Early Treatment Diabetic Retinopathy Study (ETDRS) grading of diabetic retinopathy (DR) confirmed.

The hospital ethical committee approved this study and informed consent was taken from all the subjects.

### Methods

#### Drug Preparation

**Figure 1:**
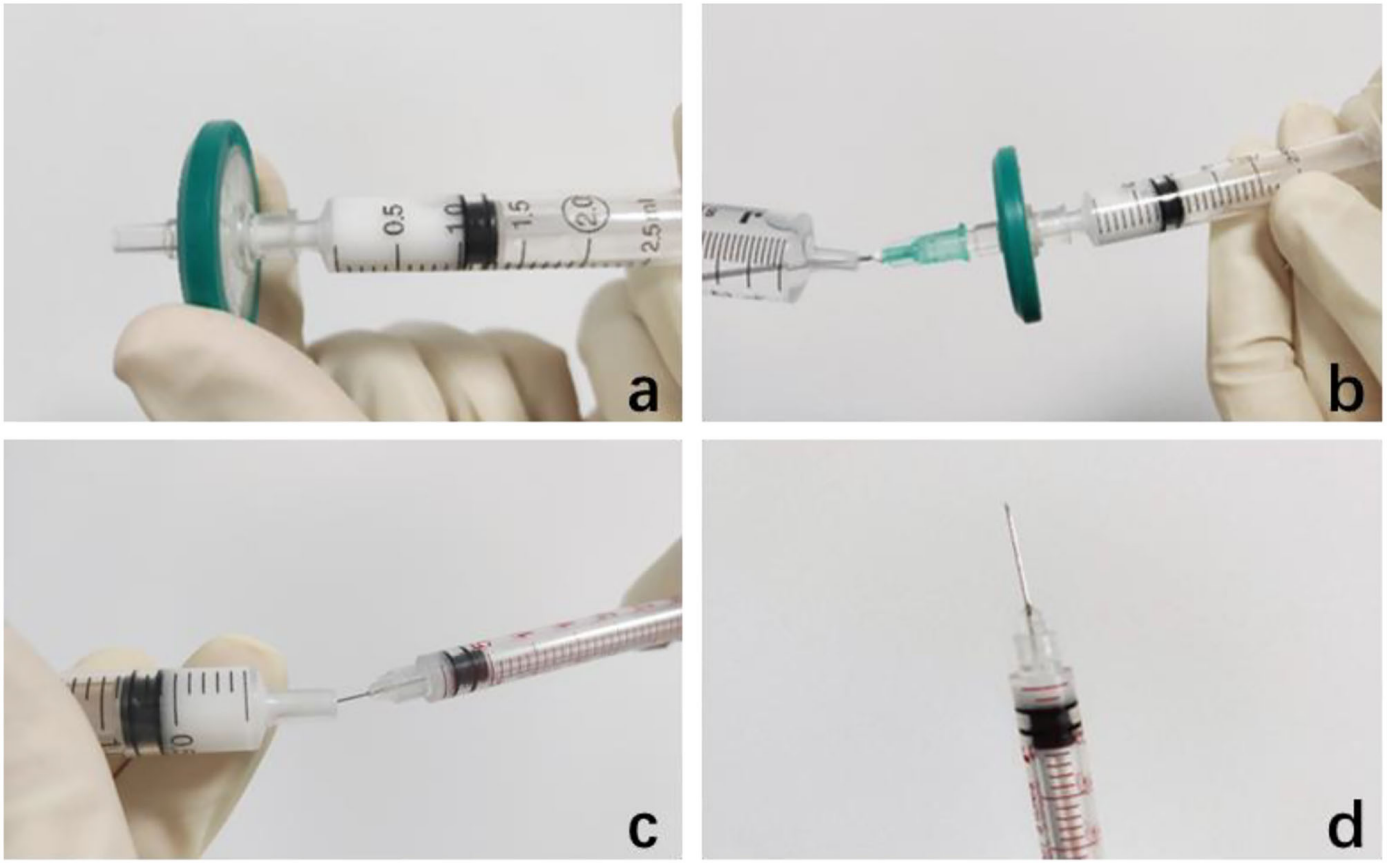
Preparation of 2 mg/0.05 ml triamcinolone acetonide (TA) injection. **(a)** Connect a filter with a diameter of 0.22 μm at the front end of the needle, push off the excipients in the suspension, and keep the TA particles on the filter membrane. **(b)** Draw 2 ml of eye-balanced salt solution to rinse it twice. **(c,d)** Transfer the 2 mg/0.05 ml TA solution into 29 G insulin syringe for use.

Out of the original ampoule (1 ml:40 mg, Zhejiang Xianju Pharmaceutical Corporation Ltd., Taizhou, Zhejiang, China), TA acetate injection was shaken and then extracted 1 ml suspension in a 2.5 ml syringe and connecting the front end of the syringe with a filter membrane with pore diameter in 0.22 μm (Millex-GP, Millipore^®^, Darmstadt, Germany, UK), after the needle was removed. Along with the suspension passing through the filter, TA particles remained on the filter membrane. The filter was rinsed twice with eye-balanced salt solution (BSS) to remove the excipients to the maximum extent. The front end of the filter is connected with the needle head to extract 2 of 1 ml of BSS through the filter to the syringe to prepare the final TA solution (2 mg/0.05 ml). Transfer the TA solution into 29 G insulin syringe for use (Figure 1).

#### Injection Method

The conjunctival sac was first soaked in 5% povidone-iodine for 90 s. Then, washed thoroughly with normal saline and later sanitized. A 29-G insulin syringe was used to puncture the sclera at 3.5 mm from the infratemporal corneal limbus. 2 mg/0.05 ml TA or 0.5 mg/0.05 ml ranibizumab (Lucentis, Novartis Pharma Stein AG, Schaffhausen, Switzerland, UK) was injected into the vitreous. After pulling out the needle, the injection point was gently pressed with a cotton swab. Subsequently, the light perception was confirmed and IOP was roughly estimated by digital tonometry on cornea. The eye was wrapped with levofloxacin gel and patients were advised to instill one drop of levofloxacin eye drops into the injected eye four times a day for 1 week after the procedure.

#### Observation Index

BCVA, CMT, IOP, times of repeated injections, and local and systemic complications were observed before injection and at 2 weeks, 1 month, 3 months, and 6 months after TA or ranibizumab injection. The Snellen visual acuity chart was used for visual acuity examination and converted to the logarithm of the minimum angle of resolution (logMAR). The macular images were collected by Heidelberg OCT (HRT-IV, TR-KT-2736, Germany, UK) fellow function. The macular fovea was scanned horizontally and vertically by the same professional. The vertical distance between the inner limiting membrane of the fovea and the inner interface of retinal pigment epithelium (RPE) was measured as CMT and a Goldman tonometer was used for monitoring IOP.

#### Statistical Analysis

The SPSS software version 22.0 (SPSS Incorporation, Chicago, Illinois, USA) was used and the differences between data of before and different time points after injection were compared by generalized estimating equations for BCVA and CMT and two-way repeated measurement ANOVA for IOP; the difference between the two groups in these three indexes was compared by least significant difference and the injection repetition rates were compared by the Fisher's exact test; median (P25, P75) was used when the measurement data do not meet the parameter test conditions. *P* < 0.05 was considered as statistically significant.

## Results

### Best-Corrected Visual Acuity

After intravitreal injection of TA or ranibizumab, BCVA at each time point of follow-up was all significantly improved compared with that of before injection. BCVA of the TA group was significantly better than that of the ranibizumab group at 3 months, not in 2 weeks and 1 month after injection (*P* < 0.05; Table 1). While one patient in the ranibizumab group still had macular edema at 1 month after injection, repeated injection was needed to get improved BCVA.

**Table 1:** Effects of intravitreal injection of triamcinolone acetonide (TA) and ranibizumab on best-corrected visual acuity (BCVA), central macular thickness (CMT), and intraocular pressure (IOP) in patients with pseudophakic cystoid macular edema (PCME).

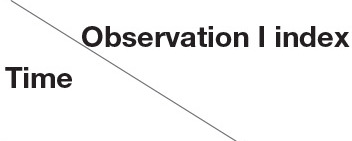	**BCVA (log MAR)**		***P***	**CMT (μm)**		***P***	**IOP (mmHg)**		***P***
	**TA**	**Ranibizumab**		**TA**	**Ranibizumab**		**TA**	**Ranibizumab**	
Before injection	0.80 (0.70, 0.96)	0.80 (0.70, 1.00)	>0.05	578.00 (469.00, 620.00)	566.00 (446.00, 634.00)	>0.05	14.68 ± 1.92	15.02 ± 2.36	>0.05
2 weeks after injection	0.30 (0.20, 0.30)^*^	0.30 (0.30, 0.40)^*^	>0.05	240.00 (219.50, 250.00)^*^	237.00 (219.00, 298.00)^*^	>0.05	14.74 ± 1.72	15.12 ± 2.70	>0.05
1 month after injection	0.20 (0.10, 0.22)^*^	0.20 (0.10, 0.30)^*^	>0.05	206.00 (193.50, 221.00)^*^	220.00 (213.00, 251.00)^*^	>0.05	16.08 ± 1.94	15.48 ± 2.02	>0.05
3 months after injection	0.10 (0.10, 0.20)^*^	0.20 (0.10, 0.40)^*^	<0.05	205.00 (191.00, 215.00)^*^	220.00 (207.00, 466.00)^*^	<0.05	15.74 ± 1.98	15.06 ± 2.41	>0.05
6 months after injection	0.10 (0.10, 0.15)^*^	0.10 (0.10, 0.20)^*^	>0.05	200.00 (191.50, 214.00)^*^	215.00 (202.00, 223.00)^*^	>0.05	15.36 ± 1.84	14.35 ± 2.39	>0.05
Wald χ^2^/F	941.858	223.683		709.391	250.192		1.394	0.721	
*P*	<0.05	<0.05		<0.05	<0.05		>0.05	>0.05	

* *There was a significant difference compared with that before injection (P < 0.05)*.

In the TA group, macular edema recurred in 1 patient with DM at 3 months after injection and decreased BCVA was significantly improved with repeated IVTA. In the ranibizumab group, four patients with DM had macular edema relapse at 3 months after injection, who need repeated injection to get improved BCVA. These indicated that DM could be a confounding factor, so we further stratified patients into subgroups, with or without DM. The statistical analysis showed that the significance at 3 months in the TA group only existed in patients with DM (Table 2).

**Table 2:** Stratified analysis for patients with or without diabetes mellitus (DM) analysis in BCVA, CMT, and IOP.

	**BCVA**						**CMT**						**IOP**					
	**With DM**			**Without DM**			**With DM**			**Without DM**			**Without DM**			**Without DM**		
	**TA**	**Ranibizumab**	***P***	**TA**	**Ranibizumab**	***P***	**TA**	**Ranibizumab**	***P***	**TA**	**Ranibizumab**	***P***	**TA**	**Ranibizumab**	***P***	**TA**	**Ranibizumab**	***P***
Before injection	0.65 (0.58, 1.055)	0.80 (0.70, 1.00)	>0.05	0.80 (0.70, 0.92)	0.80 (0.70, 1.00)	>0.05	610.50 (531.75, 649.00)	609.00 (446.00, 634.00)	>0.05	577.00 (459.00, 612.00)	510.5 (422.25, 617.00)	>0.05	15.55 ± 1.57	14.76 ± 2.90	>0.05	14.41 ± 1.98	15.25 ± 1.96	>0.05
2 weeks after injection	0.30 (0.175, 0.325)	0.40 (0.30, 0.40)	>0.05	0.22 (0.20, 0.30)	0.30 (0.225, 0.30)	>0.05	241.00 (240.00, 263.25)	298.00 (230.00, 350.00)	>0.05	230.00 (211.00, 249.00)	233.00 (216.00, 240.75)	>0.05	16.20 ± 1.46	15.26 ± 3.15	>0.05	15.60 ± 1.81	15.00 ± 2.46	>0.05
1 month after injection	0.21 (0.15, 0.22)	0.20 (0.10, 0.40)	>0.05	0.20 (0.10, 0.22)	0.20 (0.125, 0.275)	>0.05	225.00 (210.25, 245.25)	237.00 (220.00, 253.00)	>0.05	198.00 (190.00, 220.00)	215.50 (209.50, 220.75)	>0.05	15.90 ± 1.79	16.04 ± 2.42	>0.05	16.14 ± 2.04	14.99 ± 1.61	>0.05
3 months after injection	0.20 (0.075, 0.27)	0.40 (0.20, 0.50)	<0.05	0.10 (0.10, 0.20)	0.10 (0.10, 0.175)	>0.05	191.00 (187.5, 247.25)	466.00 (226.00, 498.00)	<0.05	209.00 (200.00, 216.00)	208.50 (203.25, 218.50)	>0.05	15.03 ± 1.87	15.50 ± 2.76	>0.05	15.96 ± 2.02	14.69 ± 2.18	>0.05
6 months after injection	0.16 (0.00, 0.22)	0.20 (0.10, 0.30)	>0.05	0.10 (0.10, 0.10)	0.10 (0.025, 0.175)	>0.05	214.00 (209.50, 227.25)	223.00 (215.00, 246.00)	>0.05	197.00 (190.00, 203.00)	208.50 (199.50, 217.25)	>0.05	15.65 ± 1.87	14.07 ± 1.94	>0.05	15.27 ± 1.87	14.60 ± 2.83	>0.05
Wald χ^2^/F	1,106.482	104.218		492.557	872.826		950.792	145.803		292.986	799.477		0.510	0.706		2.460	0.242	
*P*	<0.05	<0.05		<0.05	<0.05		<0.05	<0.05		<0.05	<0.05		>0.05	>0.05		>0.05	>0.05	

### Central Macular Thickness

In both the TA and ranibizumab groups, mean CMT at each time points after injection was significantly lower than that of before injection (*P* < 0.05). The mean CMT of the TA group was lower than that of the ranibizumab group from 1 month after injection and there was a significant difference between the two groups at 3 months after injection (*P* < 0.05; Table 1).

**Figure 2:**
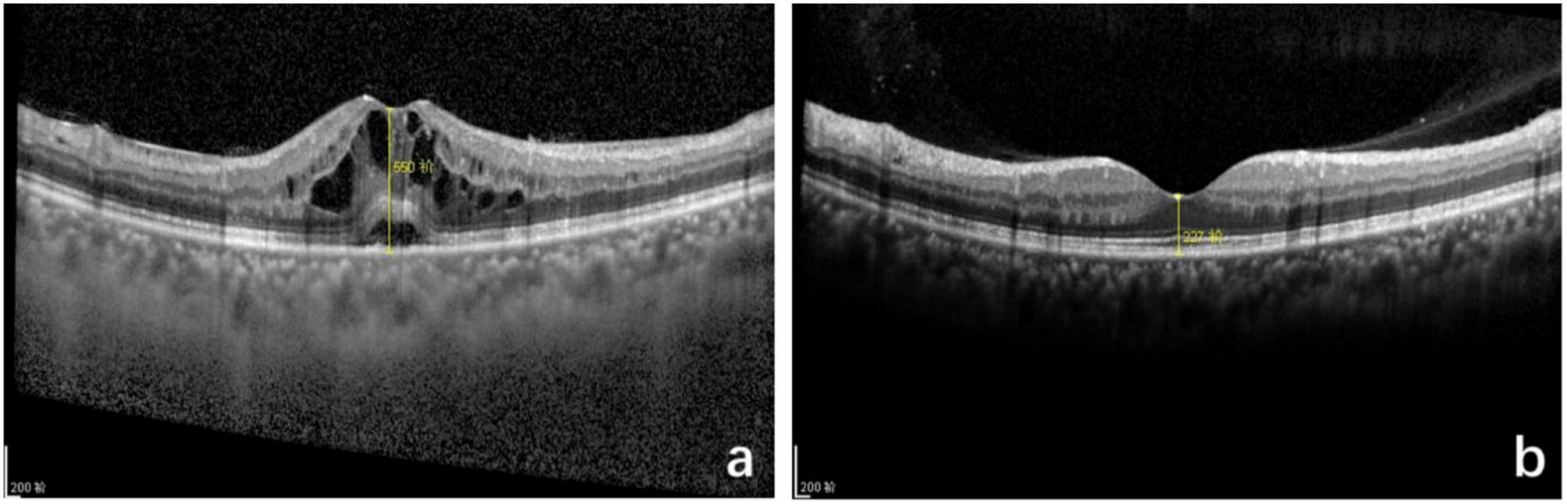
Optical coherence tomography (OCT) macular image before and after intravitreal TA treatment of pseudophakic cystoid macular edema (PCME). **(a)** Before injection, cystoid macular edema, central macular thickness (CMT): 550 μm. **(b)** Six months after injection, standard macular structure, CMT: 227 μm.

In the TA group, one patient with DM, CMT decreased significantly at 1 month, but increased again at 3 months after injection; fundus fluorescein angiography revealed capillary leakage. Second dose of IVTA was given, followed by fundus laser treatment and CMT remained normally at 6 months after injection. Other 24 patients had stable CMT at 1, 3, and 6 months after injection. Representative OCT image are showed in Figure 2.

In the ranibizumab group, 1 patient still had macular edema after 1 month of surgery and after repeated intravitreal injection, CMT decreased significantly and remained stable at 3 and 6 months follow-up. In four patients with DM, CMT decreased at 1 month and increased again at 3 months after injection. After repeated intravitreal injection of anti-VEGF, CMT decreased significantly and remained stable during the follow-up period.

Similar to BCVA, the better anesis of CMT in the TA group at 3 months after injection only existed in patients with DM (Table 2).

### Intraocular Pressure

Intraocular pressure of all the patients at each time point after the operation was in the normal range (<21 mm Hg). There was not significant difference in IOP between before and after injection, and between the two groups at any follow-up visit (*P*>0.05; Table 1), so did the stratified analysis for patients with or without DM.

### Repeated Injections

Out of 25 eyes in the TA group, only one eye had increased CMT at 3 months follow-up and needed a repeated injection, accounting for 4%. There were 15 eyes in the ranibizumab group and five patients required repeated injection, accounting for 33.3%.

The stratified analysis showed that there was no repeated injection in the TA group and 12.5% in the ranibizumab group for patients without DM, while 16.7% in the TA group and 57.1% in the ranibizumab group required repeated injection for patients with DM, which had a significant difference.

### Complications

There was no hemorrhage, endophthalmitis, retinal detachments, and other complications observed during 6 months follow-up.

## Discussion

For more than 50 years, PCME has been considered an important cause of compromised vision after cataract surgery. It has a certain degree of self-limitation, but the degree of visual impairment in some patients continues to worsen postoperatively, even if the final macular edema subsided, the macular foveal or parafoveal retinal structure has changed, causing permanent visual impairment. Therefore, we performed active treatment for patients who failed to respond to conservative treatment for 1 month. The pathogenesis of PCME is not clear, but inflammation is considered to be the leading cause.

Triamcinolone acetonide is a synthetic long-acting glucocorticoid that is difficult to dissolve in water and can be absorbed slowly in local applications. It has strong anti-inflammatory and anti-allergic effects and can reduce nonspecific inflammatory reactions such as exudation, edema, and telangiectasia. Subtenon and peribulbar injection of TA for the treatment of PMCE has already been reported (17). In the vitreous cavity pathway, drugs act directly on the retina and, hence, it is a faster and more significant route (12). Previous literature has reported that TA intravitreal injection of 4 mg/0.1 ml can significantly improve vision and macular edema, but 49–53% of patients have increased IOP than baseline preoperative data. It is recommended that long-term monitoring of IOP should be performed at least 6 months after injection (18, 19). Baseline IOP > 16 mm Hg (18), DM (19), and multiple injections are the risk factors of raised IOP. Another study of PCME treatment with TA (4 mg/0.1 ml) in 14 eyes, IOP was significantly increased compared to the baseline after 3 and 6 months follow-up. VA improved during 12 months of follow-up and CMT was significantly decreased after a single dose of injection (12). To determine the optimal dose of IVTA, a study was conducted by using 1, 2, and 4 mg doses of IVTA injection to treat DME. A trend of higher IOP and worsening CMT was seen in the 4 mg group and there was no advantage of using higher doses over the lower 1 and 2 mg doses. To decrease the adverse effects of the higher doses of IVTA, some physicians have started to use lower doses of IVTA to avoid IOP elevation (20).

Storey et al. (21) used 2 mg IVTA suspension (*n* = 106) in patients with different retinal pathologies. After a single injection of IVTA, 10.4% of patients had IOP rise more than 10 mm Hg. Compared to their baseline of IOP, 13.2% of patients after a median of 1.6 injections had increased IOP with an average peak of 29 mm Hg (21). Incidence of elevated IOP was still higher with 2 mg TA than our findings with filtered modified 2 mg TA. Although a lower dose of TA has been used, unlike this study, TA was original commercial suspension and it was not modified by filter. At present, most of the commercially available TA is a suspension (12, 20, 21). Many components in excipients can cause the decrease or even loss of photoreceptors cells in the retina and it may be related to the dose (22). Some investigators have found that benzyl alcohol present in the commercially available TA suspension could be toxic for intraocular tissues, which can raise IOP (23, 24), Hong et al. (25) reported that patients receiving IVTA injections containing a preservative showed significantly elevated IOP (25.8%) as compared to those who received preservative-free IVTA (9.1%). Garcia-Arumi and his associates (26) explained that filtration techniques by using a 0.22 or 5-μm pore size membrane could reduce the possible toxicity of benzyl alcohol in commercially available TA suspension (Trigon Depot, 40 mg/ml, Bristol-Myers). The membrane pore size inversely influenced the final concentration of TA. We used a 0.22-μm pore size filter membrane to remove the influence of excipients to a greater extent. In addition, the dosage of TA injection was only 2 mg/0.05 ml and half of that reported in the previous literature for PCME. After the procedure, BCVA was improved significantly and macular edema was subsided. Complications such as elevated IOP, hemorrhage, endophthalmitis, and retinal detachments were not observed during 6 months of follow-up. Hence, it is suggested that the intravitreal injection of filtered modified 2 mg TA is safe and more effective for PCME.

Anti-VEGF can inhibit the formation of ocular neovascularization, reduce vascular permeability, leakage, and reduce CME caused by angiogenic ophthalmopathy (2). It has been reported that intravitreal injection of anti-VEGF is effective for PCME and IOP is normal during follow-up (27, 28). Lim et al. (14) found that intravitreal injection of 4 mg TA and 1.25 mg BVB could improve the VA of diabetic patients with macular edema. After 6 months of follow-up, CMT reduction in the TA group was more than the BVB group. The ratio of the patient for requiring repeated injections was significantly lower in the TA group (16.7% in the TA group and 70.6% in the BVB group). It may be related to the fact that TA cannot only resist VEGF, but can also fight against other inflammatory factors. It also reduces the breakdown of the blood-retinal barrier, prevents the production of proinflammatory prostaglandins, and also controls the production of VEGF (29). Our findings also support this because only TA maintained a sustained reduction in CMT over a 6-month period. Although the significant difference was only at 3 months, a trend toward a greater improvement in BCVA and CMT after surgery in the TA group as compared to the ranibizumab group. The rate of retreatment injection was also low in the TA group (4%) compared to the ranibizumab group (33%). For patients with DM, 16.7% in the TA group and 57.1% in the ranibizumab group required repeated injection; it means that IVTA is more effective in patients with PCME with diabetes. There were no side effects of a rise in IOP after IVTA injection because we used filtered modified 2 mg TA.

Ranibizumab is a low molecular mass anti-VEGF agent. It can penetrate deep into the retina, crossing the blood-retinal barrier, and is easy to be absorbed. In this research, BCVA and CMT are still better in the TA group over 6 months follow-up than the ranibizumab group. Also, for patients with PCME with vitreous traction or epiretinal membrane, anti-VEGF agent treatment is ineffective and has the risk of aggravating retinal traction (2). TA has the effect of promoting posterior vitreous detachment and has no interference with the macular epiretinal membrane, making it safer. Anti-VEGF drugs are also expensive, increasing the medical expenses of the patient. Although the risk of raised IOP with anti-VEGF injections is not commonly reported, a recent large pharmacoepidemiologic study reported that seven or more injections of BVB annually increase the risk of requiring glaucoma surgery (30).

Dexamethasone (DEX) vitreous implant (such as Ozurdex) is a new self-degradable glucocorticoid sustained-release system. Previous studies have confirmed that it significantly affects relieving PCME (31, 32). But, the need for monitoring, repeated injections, and its high price limit its clinical application. Furthermore, the risk of high IOP and macular edema recurrence still cannot be avoided. Intravitreal injection of sterile-filtered 4 mg TA and 0.7 mg DEX implant (Ozurdex) is equally effective in increasing BCVA in patients with PCME at a 6-month follow-up. However, it seems that the macular edema responds more rapidly and is significantly superior in the TA group and CMT reduction reached maximum effect only 1 week after injection (33). Our results also confirmed that IVTA achieved increasing BCVA and CMT reduction from 2 weeks after injection, without obvious increase in IOP.

## Conclusion

In conclusion, our findings have shown that both the TA and ranibizumab intravitreal injection can achieve in improved BCVA and reduced CMT in patients with PCME, with a trend toward greater improvement in the TA group and the difference was significant at 3 months. Further stratified analysis showed that the therapeutic effect of the TA group was significantly better than that of the ranibizumab group at 3 months after injection in patients with DM, while not in patients without DM.

Intravitreal injection of filtered modified 2 mg TA is safe, effective and an inexpensive alternative to anti-VEGF agents for patients with PCME, especially for patients concurrently with DM. However, a large number of clinical randomized controlled studies along with long-term follow-up observations are needed.

## Data Availability Statement

The raw data supporting the conclusions of this article will be made available by the authors, without undue reservation.

## Ethics Statement

The studies involving human participants were reviewed and approved by the Medical Ethics Committee of the Second Affiliated Hospital of Xian Jiaotong University. Written informed consent to participate in this study was provided by the participants' legal guardian/next of kin. Written informed consent was obtained from the individual(s) for the publication of any potentially identifiable images or data included in this article.

## Author Contributions

LB and SZ designed the study. FT and YW wrote the original draft. LB carried out the surgery. YH and FT carried out the measurements and follow-ups. YW and LB together with SZ and BM analyzed the results and further revised the manuscript. All authors have read and approved the final version of the manuscript.

## Funding

This study was supported by the Research and Development Program of Shaanxi Province (No. 2021SF-161) and the Medical Research Project of Xi'an Science and Technology Action Plan [2019114613YX001SF041 (1)]. The funders had no role in the study design, data collection, analysis, or manuscript writing.

## Conflict of Interest

The authors declare that the research was conducted in the absence of any commercial or financial relationships that could be construed as a potential conflict of interest.

## Publisher's Note

All claims expressed in this article are solely those of the authors and do not necessarily represent those of their affiliated organizations, or those of the publisher, the editors and the reviewers. Any product that may be evaluated in this article, or claim that may be made by its manufacturer, is not guaranteed or endorsed by the publisher.
